# Does radiofrequency ablation (RFA) epiphysiodesis affect adjacent joint cartilage?

**DOI:** 10.1007/s11832-016-0747-3

**Published:** 2016-06-09

**Authors:** Juan Manuel Shiguetomi-Medina, O. Rahbek, A. A. H. Abood, H. Stødkilde-Jørgensen, J. L. Ramírez Garcia-Luna, B. Møller-Madsen

**Affiliations:** Orthopaedic Research Laboratory, Danish Paediatric Orthopaedic Research, Aarhus University Hospital NBG, Aarhus University, Noerrebrogade 44 Building 1A, 8000 Aarhus C, Denmark; Department of Children’s Orthopaedics, Aarhus University Hospital NBG, Noerrebrogade 44, 8000 Aarhus C, Denmark; The MR Research Center, Aarhus University Hospital, Skejby, Brendstrupgårdsvej 100, 8200 Aarhus N, Denmark; Department of Clinical Epidemiology and Public Health, Facultad de Medicina, Universidad Autónoma de San Luis Potosí, Venustiano Carranza 2045, 78210 San Luis Potosí, SLP Mexico

**Keywords:** Epiphysiodesis, RFA, MRI, Cartilage damage

## Abstract

**Purpose:**

To test the hypothesis that epiphysiodesis made with radiofrequency ablation (RFA) is a safe procedure that disrupts the growth plate without damaging the adjacent joint articular cartilage.

**Methods:**

RFA epiphysiodesis was done during 8 min in vivo in 40 growing pig tibia physis. In addition, three tibiae were ablated for 16 min and three more for 24 min. As a burned cartilage reference, six tibiae were ablated on the joint articular cartilage for 8 min. After the procedure, the animals were terminated and the tibiae were harvested. Magnetic resonance imaging (MRI) was done ex vivo to evaluate the joint articular cartilage in all samples. We used T1-weighted, T2-weighted, and water content sequences under a 1.5 T magnetic field.

**Results:**

On the burned articular cartilage, intensity changes were observed at MRI. We found no evidence of articular cartilage damage on the 40 8-min RFA procedures. The tibiae ablated for 16 min and 24 min showed intact joint cartilage.

**Conclusions:**

Epiphysiodesis using RFA is safe for the adjacent articular cartilage. This study shows that RFA can be done safely in the growing physis of pigs, even with triple duration procedures.

## Introduction

Cartilage has a nonlinear thermomechanical behavior [1]. Díaz et al. report cartilage changes at increasing temperatures: at 50 °C, thermal injury is observed; when 56 °C is reached, there is a sharp decrease in cell variability associated to compromised cell membranes, which lead to cell death at 60 °C [2]. Chondrocytes are particularly sensitive to temperature. Articular cartilage damage is permanent and progressive. Degeneration and swelling of the cartilage reduce its ability to absorb compressive load, resulting in crepitus and pain [3]. Chondral defects are seen in 60 % of all knee arthroscopy procedures with injury to articular cartilage, causing progressive and permanent damage. Heat starts decreasing glycosaminoglycan, metalloproteinase 13, interleukin 1, and nitric oxide, which cause significant changes to the cartilage [4]. Chondrocyte death in only one-fourth of the articular cartilage thickness causes irreversible, extended damage [5]. Epiphysiodesis using radiofrequency ablation (RFA) has been reported to be successful in animal models. It can induce growth arrest in both small [6] and large species [7]. This procedure induces thermal damage and coagulates necrosis in the physis. Because of the proximity of the physis and the epiphysis, there is a theoretical possibility to damage the articular cartilage when performing epiphysiodesis using RFA. Ablation devices have been deployed in treatment settings requiring tissue preservation, like debridement chondroplasty and modeling. Because of this, several articular cartilage models have been designed to study tissue preservation and perturbation [8]. The risk of damaging the adjacent joint articular cartilage when performing epiphysiodesis using RFA has not been reported, to our knowledge.

Aim: to test the hypothesis that epiphysiodesis made with RFA is a safe procedure that disrupts the growth plate without damaging the adjacent joint articular cartilage.

## Methods

### Ethics

The study was conducted in compliance with the Danish Animal Research Guidelines. Ethical Committee authorization was not needed; this was an acute, nonsurvival study.

### Design

The study was divided into four sub-studies: positive controls (burned cartilage), study A (8-min-long ablation, 20 animals, 40 bilateral procedures), study B (16-min-long ablation, three animals, six bilateral procedures), and study C (24-min-long ablation, three animals, six bilateral procedures).

#### Burned cartilage reference parameters

The proximal tibia (plateau) articular cartilage was ablated for 8 min in six animals (12 bilateral ablations). The obtained data were used as reference for damaged cartilage. The procedure was done laterally and medially.

#### Physis RFA

Ablation was done directly in the tibia physis for 8 min (study A), 16 min (study B), and 24 min (study C). The procedure was done laterally and medially.

### Sample size

For study A, we expected an intact articular cartilage in the adjacent joint to the ablation (tibiae plateau) visible on magnetic resonance imaging (MRI) in 100 % of the cases. The desired power was 90 % and *α* = 0.05. Thus, 20 animals were included in the study. Two procedures were done on each animal (medial and lateral ablation). In addition, six animals were included as positive controls (burned cartilage) and six were included for studies B (three bilateral ablations) and C (three bilateral ablations). Study B represents a double-time ablation (16 min) and study C a triple-time ablation (24 min).

### Animals

Healthy, skeletally immature female pigs from the Yorkshire–Landrace–Duroc race were used in all the procedures. All the animals were 15 weeks old and 40.2 kg on average (37.6–44.8 kg).

### Methods

#### Magnetic resonance

Images were obtained ex vivo at room temperature (21 °C). All the treated tibiae were harvested after the procedure and kept frozen at −18 °C. All MRI was done at the MR Research Center, Aarhus University Hospital, Skejby. A whole-body scanner (Siemens Magnetom Avanto 1.5 Tesla, Erlangen, Germany) was used to acquire the following four image sets sequentially:

1. Spin echo (SE) T1-weighted imaging using the following parameters: echo time (TE) 20 ms, repetition time (TR) 800 ms, slice thickness 3 mm, field-of-view (FOV) 120 × 120, matrix 384 × 268, and two signal averages (NEX).
2. Water content in the tissue was calculated from T1 maps. Both the two flip angle method and inversion recovery sequences were applied. The two flip angle method (gradient echo sequence with flip1 5°, flip2 30°, TE 1.61 ms, TR 15 ms, slice thickness 4 mm, NEX 3, FOV 180 × 101 mm, and matrix 256 × 256) is fast, with an acquisition time of 14 min. The inversion recovery method (11 inversion times from 200 to 2200 ms, slice thickness 4 mm, FOV 200 × 200, matrix 256 × 256) has an acquisition time of 34 min.
3. Proton density weighted fast SE sequence (TE 41 ms, TR 2000 ms, echo train length 7, slice thickness 2 mm, FOV 180 × 101 mm, matrix 448 × 314, NEX 2).
4. T2-weighted fast SE sequence (TE 73 ms, TR 4610 ms, echo train length 1, slice thickness 2.0 mm, FOV 160 × 130 mm, matrix 256 × 192, NEX 6).

#### Anesthesia and medication

For premedication, the animals received an intramuscular injection with ketaminol (5 mg/kg; S-ketamine, Pfizer, Berlin, Germany) and midazolam (0.5 mg/kg; Hypnomidate, Janssen-Cilag, Beerse, Belgium). The animals were intubated and anesthesia was maintained with a gas mixture of sevoflurane (Sevorane, Abbott Laboratories, Chicago, IL, USA) and O_2_ 60 %. For analgesia, an intravenous infusion of fentanyl (0.025 mg/kg/h; Haldid, Janssen-Cilag, Beerse, Belgium) was maintained during the procedure.

#### Epiphysiodesis

All the procedures were done using a 7-mm, 18 G radiofrequency probe without cooling (Cool-tip™ RFA system, Covidien AG, USA). Ablation power (watts) was corrected constantly during the procedure to maintain the temperature between 92 and 98° C. Impedance fluctuated between 90 and 700 Ω during the procedure.

#### Operation technique

**Fig. 1:**
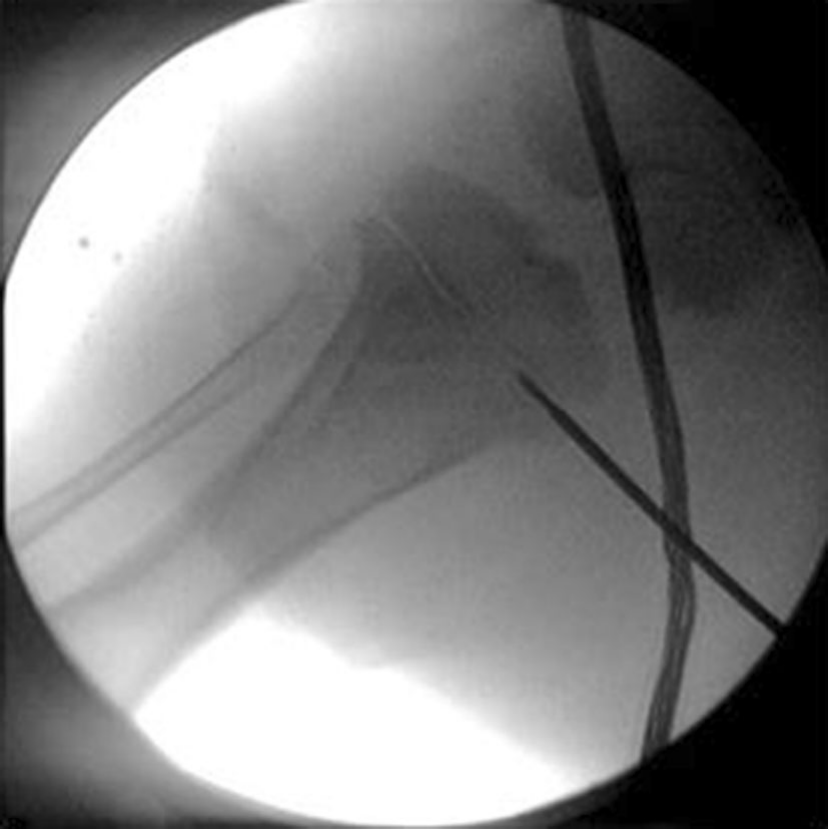
Fluoroscope image. The growth plate was identified and a radiofrequency (RF) probe inserted into the physis to perform epiphysiodesis

Under fluoroscopic guidance (Fig. 1), the proximal tibiae growth plate was identified and a penetration cannula (Bonopty^®^ Bone Biopsy System, AprioMed AB, Uppsala, Sweden) was inserted 90° from the vertical plane towards the growth plate. From the skin through the soft tissue, a stylet was used and when the periosteum was reached, it was drilled into the growth plate. Then, the radiofrequency probe was inserted 1 cm into the growth plate and the ablation was done.

#### Euthanasia

After the ablation, under general anesthesia, the animals received a lethal intravenous injection of pentobarbital 0.5 ml/kg (200 ml/kg) and the tibiae were harvested.

### Analysis

**Fig. 2:**
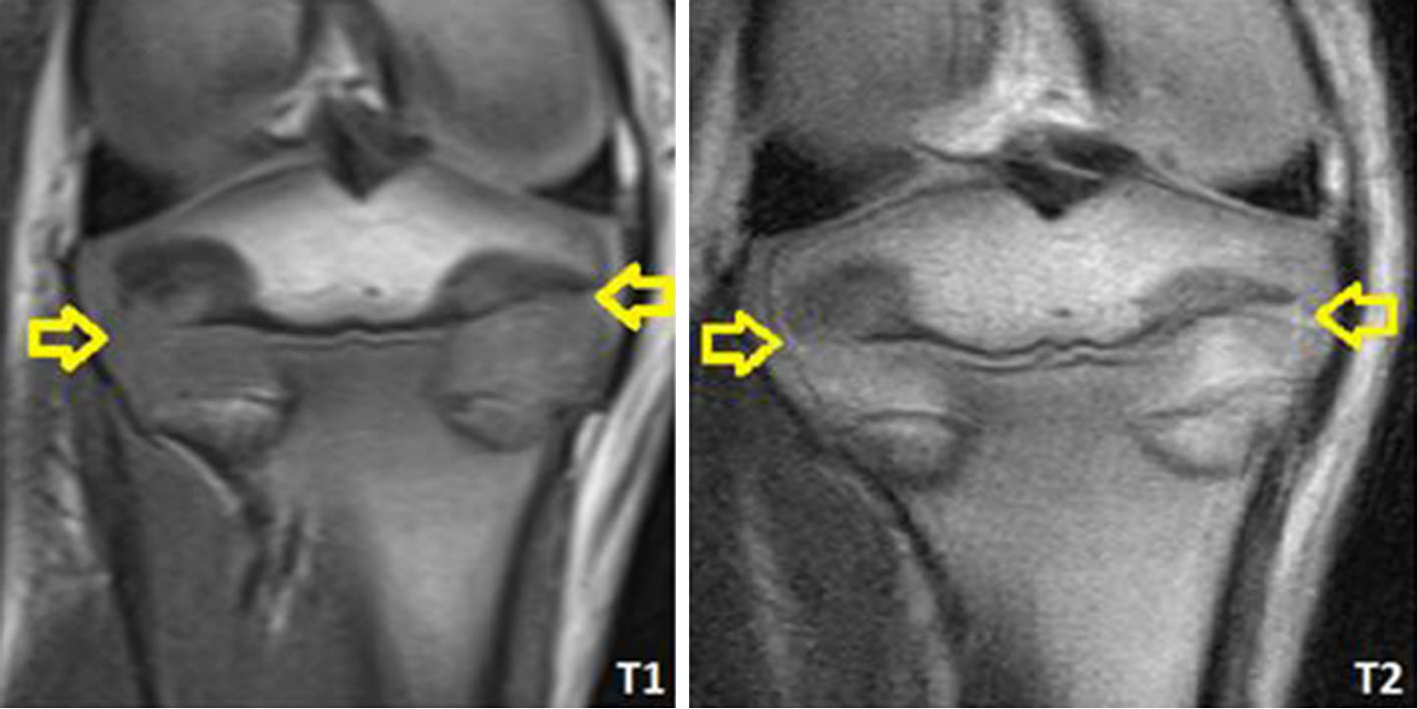
Magnetic resonance images (T1 and T2), study A. After the procedure, the physis can be observed to be disrupted (*arrows*). The articular cartilage above the epiphysiodesis looks continuous and homogenous

**Fig. 3:**
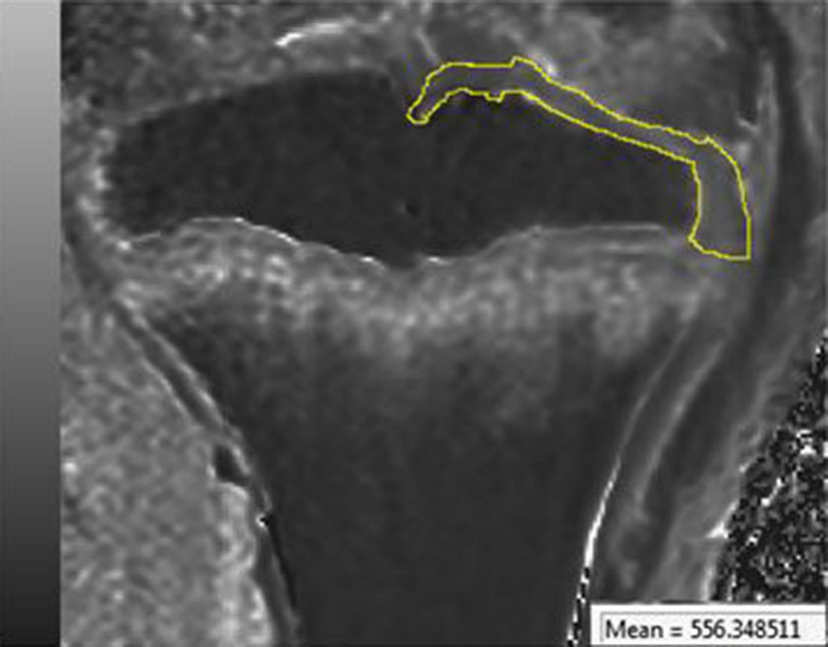
Water content calculation. An area of interest was established around the joint cartilage (*yellow*) and a mean T1 value was obtained. This allows calculating the water content of this area

Magnetic resonance images were analyzed using Syngo FastView software (Siemens©, AG, Berlin and Munich 2004–2008). The proximal tibial joint cartilage was identified and then analyzed for discontinuity and intensity changes (Fig. 2). Also, the T1 value of the cartilage (Fig. 3) was measured, seeking changes above 25 % using Siswin software version 0.9 (Steffen Ringgaard© 2008).

## Results

### Physis-ablated tibiae (studies A, B, and C)

None of the ablated tibiae showed intensity changes on MRI (Fig. 2). T1 values from the articular cartilage showed a mean difference of 3 % (2–5 %) between the different MR sections. We did not find any difference between the tibiae that were ablated for 8, 16, and 24 min. All the values were similar (Table 1).

**Table 1:** T1 values. Mean values and standard deviation (SD) are shown for all the study groups

	Non ablated (*n* = 6)	8 min ablation (*n* = 40)	16 min ablation (*n* = 3)	24 min ablation (*n* = 3)	Burned (*n* = 6)
Mean T1 value	646	644	651	639	1176
SD	30	39	29	27	114

### Burned cartilage group

**Fig. 4:**
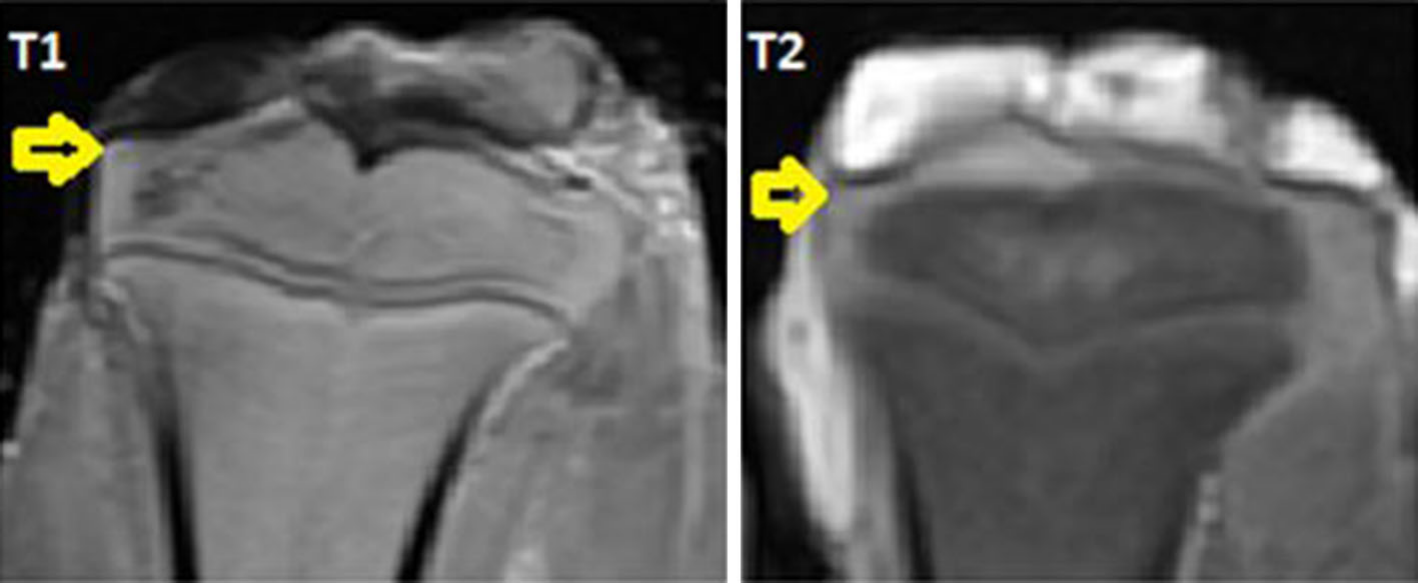
Positive controls with ablated joint cartilage. On magnetic resonance imaging (MRI), hyper-intense changes can be observed in the intentionally ablated joint cartilage (*arrows*)

**Fig. 5:**
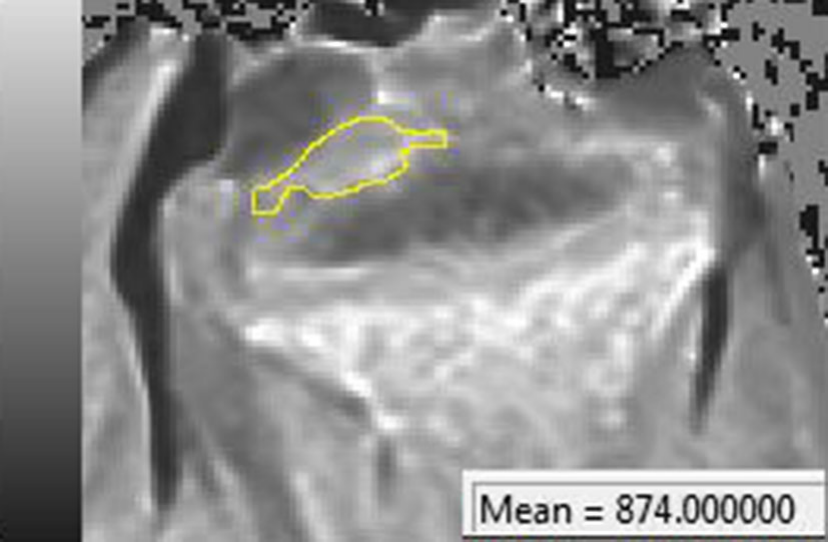
Burned cartilage water content calculation. An area of interest was established around the intentionally burned joint cartilage (*yellow*). Based on the mean T1 value, the water content was calculated

A hyper-intensity was observed at the ablation area (Fig. 4). T1 values from these areas were increased by 30 % on average (20–42 %) (Fig. 5; Table 1).

## Discussion

**Fig. 6:**
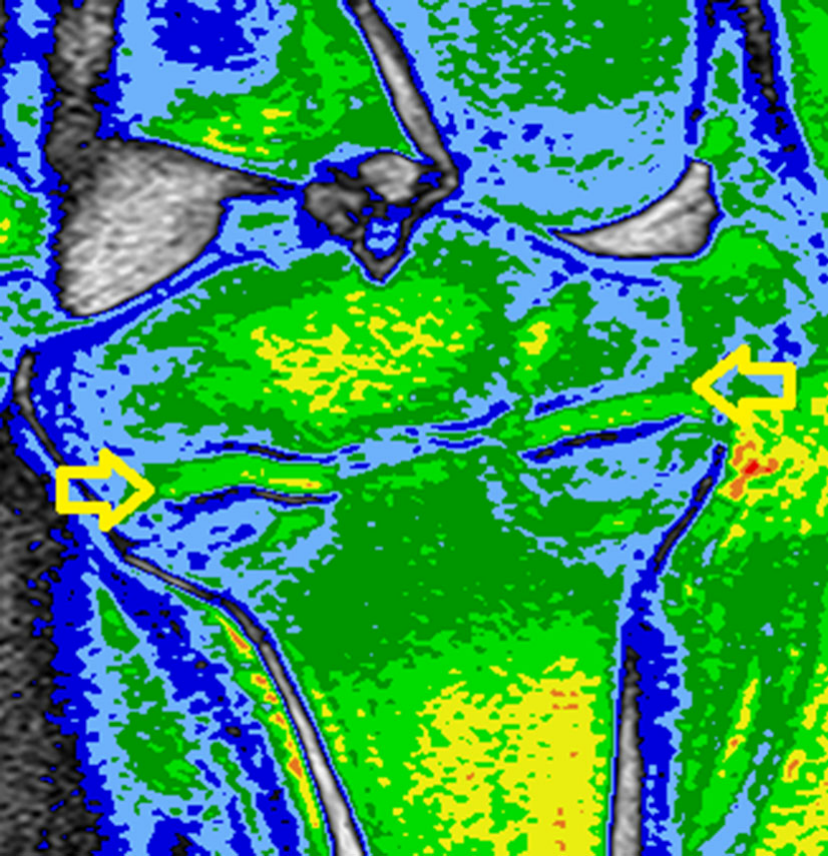
Colored water content MRI, study A. The physeal ablation sites become evident (*arrows*)

This nonsurvival study is based on a previously published protocol that results in growth arrest without giving the animal discomfort or complications during the follow-up [7]. Early changes caused by thermal damage to the articular cartilage are described by Meister et al. [9]. They report that thermal lesions of articular cartilage can be observed as “shrinkage” surrounded by edema. MR is accepted to analyze cartilage damage and it can detect early changes that result in degeneration [10]. After analysis using MR, we did not find any changes in the procedures where the physis was ablated. We could not identify shrinkage of the articular cartilage in the group that was ablated directly on the cartilage, but edema was evident and it was measured indirectly using MR T1 values [11]. In the present study, we chose a porcine model because the bone biology resembles humans and the distance between the physis and the articular cartilage is closely equal to humans [12]. Subjective evaluation of MRI both in T1-weighted and T2-weighted images can suggest tissue changes. In colored water content images (Fig. 6), these changes are easier to identify. However, an objective analysis is more accurate and can be obtained from T1 mapping. This allows the quantification of damage and usage of reference parameters. The water content allows it to be known if the cells are compromised or they suffered damage. Damaged cell membranes lead to cell death. Thermal damage affects mainly membranes. Such changes directly affect the water content immediately [3]. Damaged membranes lead to edema, which causes an influx of calcium. This phenomenon can be observed even in temperatures of 50 °C, which would not cause cellular death but would suppress the ability of chondrocytes to remodel, leading to permanent damage [13].

MR images from the physis ablated 8, 16, and 24 min looked similar on MRI, where hyper-intensities or morphological changes could not be identified. From the T1 values, we concluded that edema was not present at the articular cartilage after the procedure. An isolative effect from the bone is described by Caffey et al. [14]. On a study in fresh cartilage, they conclude that radiofrequency probes produce significant cellular death after 1 and 3 s. However, this does not happen when the probe is placed 1 mm from the cartilage. Our porcine model is close to biological human characteristics, but a validation for human tissue should be done from our translational research. Also, during childhood, the epiphysis size varies. Our model does not consider this size variation, which could be a limitation. Another limitation of our study could be that we only included coronal images, although we believe that it does not affect our analysis.

In conclusion, RFA of the tibia physis is a safe procedure for the adjacent joint cartilage. The energy and heat is contained at the ablation site. We found no evidence of joint cartilage damage based on macroscopy, MRI intensity, and water content.
